# *PHF6* mutation is associated with poor outcome in acute myeloid leukaemia

**DOI:** 10.1002/cam4.5173

**Published:** 2022-09-29

**Authors:** Kexiu Huang, Lei Wang, Yaling Zheng, Chunyan Yue, Xuedan Xu, Hongbo Chen, Rui Huang, Yuhua Li

**Affiliations:** ^1^ Department of Haematology Zhujiang Hospital of Southern Medical University Guangzhou P.R. China; ^2^ Department of Haematology Jiangmen Central Hospital JiangMen P.R. China; ^3^ School of Pharmaceutical Sciences (Shenzhen) Sun Yat‐sen University Shenzhen P.R. China

**Keywords:** acute myeloid leukaemia, haematopoietic stem cell transplantation, mutation, PDH finger protein 6, prognosis

## Abstract

**Introduction:**

Mutation of plant homeodomain finger protein 6 (PHF6) occurs in approximately 3% of acute myeloid leukaemia (AML) cases. Although it was reported to be associated with poor prognosis, it was not confirmed by other groups. Recently, propensity score matching has provided an effective way to minimise bias by creating two groups that are well balanced with respect to baseline characteristics, providing more convincing results, which has an advantage, especially for rare subtype studies. To provide further evidence on the role of PHF6 mutation, we performed a retrospective propensity score‐matched cohort study to assess the therapeutic responses and survival outcomes of AML patients with PHF6 mutation compared with those without PHF6 mutation after balancing age, sex and risk categories.

**Patients and Methods:**

A total of 22 patients with PHF6 mutation from 801 consecutive newly diagnosed AML cases in our center were identified, and 43 patients with the PHF6 wild‐type genotype were successfully matched at a 1:2 ratio.

**Results:**

AML harbouring PHF6 mutation was associated with a lower complete remission (CR) rate (41% vs. 69%; OR = 3.64, 95% CI 1.10, 12.10; p = 0.035) and shorter median overall survival (OS) (6.0 vs. 39.0 months; p < 0.001) and event‐free survival (EFS) (2.0 vs. 11.0 months; p = 0.013) compared with PHF6 wild‐type patients. Further multivariate analysis supported that PHF6 mutation was an independent risk factor for overall survival in AML (HR = 8.910, 95% CI 3.51, 22.63; p < 0.001). In addition, allogeneic haematopoietic stem cell transplantation (allo‐HSCT) seemed to ameliorate the poor prognosis of AML with PHF6 mutation in this study.

**Conclusion:**

Our data revealed that PHF6 mutation was associated with a lower chemotherapy response and shorter survival, suggesting that PHF6 mutation is a predictor of poor prognosis in AML.

## Graphical abstract

We performed a retrospective propensity score‐matched cohort study to assess the therapeutic responses and survival outcomes of AML patients with *PHF6* mutation compared with those without *PHF6* mutation after balancing age, sex and risk categories. Our data revealed that *PHF6* mutation was associated with a lower chemotherapy response and shorter survival, suggesting that *PHF6* mutation is a predictor of poor prognosis in AML.

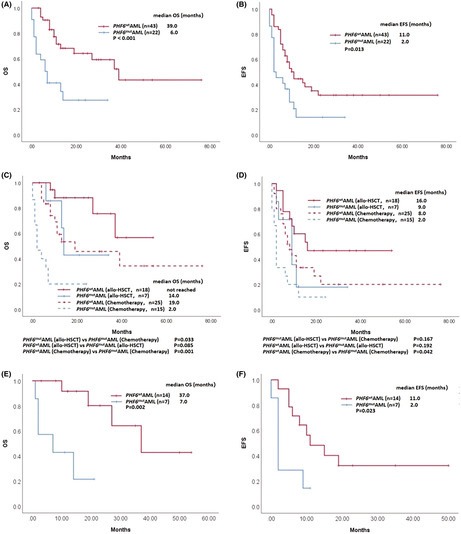

## 1 INTRODUCTION

The concept that molecular markers, in addition to cytogenetic changes, can help refine prognostic predictions for acute myeloid leukaemia (AML) has been universally accepted. Hence, identifying mutations that carry prognostic and therapeutic impacts (including *NPM1*, *FLT3*, *CEBPA*, *IDH1/2*, *DNMT3A*, *KIT*, *TP53*, *RUNX1* and *ASXL1* mutations) has become standard in diagnostic workups for AML.^1^ However, some mutations with prognostic value may not be included in current diagnostic workups due to a lack of sufficient clinical data because of their low mutation rate. Ignoring these rare gene mutations that potentially lead to adverse prognoses may result in patients who carry these mutations being assigned to favourable or moderate prognosis groups and not receiving allogeneic haematopoietic stem cell transplantation (allo‐HSCT) in first remission and thus missing the window for potentially curative treatment. Thus, identifying genes with low mutation rates that are associated with significantly poor prognoses has clinical implications.

Plant homeodomain finger protein 6 (*PHF6*) is a member of the plant homeodomain‐like finger family whose germline mutation was known to cause Börjeson‐Forssman‐Lehmann syndrome (BFLS).^2^ In addition, *PHF6* somatic mutations have been recently identified in many haematologic malignancies, including approximately 20% of T‐ALL (T‐cell acute lymphoblastic leukaemia) and 3% of AML cases.^3^, ^4^, ^5^, ^6^ *PHF6* was suggested to be a tumour suppressor gene, and its loss synergizes with leukaemic lesions to promote the onset of T‐ALL.^7^, ^8^, ^9^

Nevertheless, the mechanisms of action of *PHF6* mutations and their effect on the prognosis of AML remain unclear. Few studies have demonstrated the clinical features of AML patients with *PHF6* mutation and most of them were largely descriptive with the largest study containing only 15 AML patients harbouring *PHF6* mutation. Patel et al. reported that *PHF6* mutation was associated with adverse prognosis in a cohort of 398 young AML patients via univariate analysis; however, this finding was not significant in the multivariate analysis, which was partly because this cohort contained only 9 patients with *PHF6* mutations.^6^ Another study identified 15 AML patients with *PHF6* mutation from 366 patients with AML and found no association between *PHF6* mutation and overall survival.^10^ The above studies yielded conflicting results on the role of *PHF6* mutation in AML in terms of survival. Moreover, neither study explored a relationship between *PHF6* mutation and treatment responses to conventional chemotherapy. Thus, a more credible and detailed study of the role of *PHF6* mutation in AML is urgently needed.

Recently, propensity score matching has provided an effective way to minimise bias by creating two groups that are well balanced with respect to baseline characteristics, providing more convincing results, which has an advantage, especially for rare subtype studies.^11^ Thus, we performed a retrospective propensity score‐matched cohort study to assess the therapeutic responses and survival outcomes of AML patients with *PHF6* mutation compared with those without *PHF6* mutation after balancing age, sex and risk categories.

## 2 PATIENTS AND METHODS

### 2.1 Patients

A total of 801 patients with AML admitted between April 1, 2016 and April 1, 2021 were screened from the database of Zhujiang Hospital of Southern Medical University. All cases were definitely diagnosed as AML, and mixed phenotype acute leukaemia was excluded according to the 2016 revision to the WHO classification of myeloid neoplasms and acute leukaemia.^12^ Patients were excluded from matching if they were younger than 18 years of age at the time of the initial diagnosis, did not receive induction chemotherapy, had insufficient information to be stratified according to European LeukaemiaNet (ELN) 2017 risk categories,^13^ or had acute promyelocytic leukaemia. Eventually, 22 AML patients with *PHF6* mutation (*PHF6* ^mut^AML) and 442 AML patients without *PHF6* mutation (*PHF6* ^wt^AML) were included for matching.

This retrospective, matched‐cohort study was approved by the Ethics Committees of Zhujiang Hospital of Southern Medical University.

### 2.2 Treatment protocols

For fit de novo patients, standard‐dose cytarabine‐based regimens (idarubicin 10–12 mg/m^2^/d or daunorubicin 45–60 mg/m^2^/d, d1–3; cytarabine 100–200 mg/m^2^/d, d1–7) were given as the initial induction therapy. For unfit de novo patients, low‐dose cytarabine‐based regimens (the so‐called “CAG” regimen with or without hypomethylating agents) were given as the initial induction therapy. In addition, few patients only received hypomethylating agents as their induction regimen. Novel‐targeted drugs, such as ivosidenib, enasidenib, gilteritinib and venetoclax, were not applied because they were not available during the treatment period in this study. For patients assigned to poor prognosis groups according to risk categories, allo‐HSCT was conducted in their first remission if they had suitable donors. For patients assigned to the favourable group, four to six cycles of intermediate‐dose cytarabine (1–2 g/m^2^, q12 h, 3–5 d) followed by autologous haematopoietic stem cell transplantation were given as consolidation. For patients assigned to intermediate prognosis groups, whether to perform allo‐HSCT after receiving complete remission (CR) depends on the physician's decision and the specific condition of the patients.

### 2.3 Matching process

**FIGURE 1:**
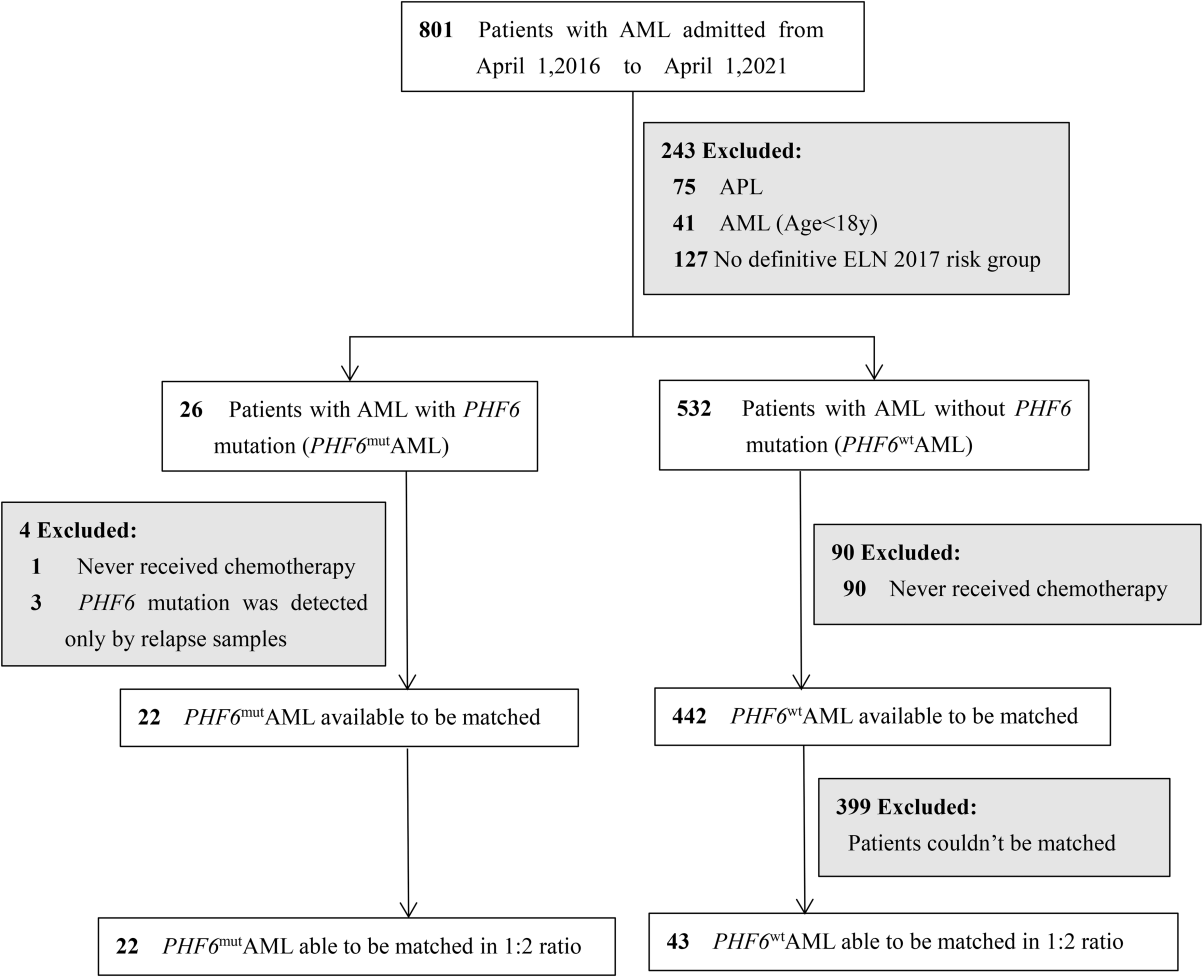
Process for matching *PHF6* ^mut^AML to *PHF6* ^wt^AML. AML, acute myeloid leukaemia; ELN, European LeukemiaNet. Paediatric AML and promyelocytic leukaemia (APL) were excluded because they are clinically different from other AML subtypes. Patients who did not receive induction chemotherapy were also excluded to ensure credible survival analysis. Age at leukaemia diagnosis, sex and risk category were used to construct the propensity score using logistic regression

To evaluate differences in survival and treatment responses, we undertook a propensity score matched analysis to compare AML patients with *PHF6* mutation with patients without *PHF6* mutation. Age at diagnosis, sex and ELN 2017 risk group were used to construct the propensity score using logistic regression. Propensity score matching was performed in a 1:2 ratio using the nearest neighbour algorithm in the MatchIt package of R with a calliper value of 0.02. Eventually, all 22 *PHF6* ^mut^AML patients were successfully matched with 43 *PHF6* ^wt^AML patients, including one *PHF6*‐mutant patient who was successfully matched with only one wild‐type control. The matching process is fully outlined in Figure 1.

### 2.4 Statistical analyses

Overall survival (OS), event‐free survival (EFS) and relapse‐free survival (RFS) were determined per the modified International Working Group criteria for AML.^14^ OS was calculated from the date of diagnosis to the date of death or last contact. EFS was measured from the date of diagnosis to date of refractory disease, relapse, death or censored at the last follow‐up. RFS was measured from the date of CR to the date of AML relapse or death from any cause.

The chi‐square test, *t* test and Mann–Whitney *U* test were used to analyse differences in the distribution of variables between different groups. The balance of matching was assessed through standardised mean differences. Univariate conditional logistic regression and odds ratios (ORs) were used to evaluate treatment effects after matching. The Kaplan–Meier method with the log‐rank test was used to compare time‐to‐event variables. Multivariate Cox regression was applied to evaluate the association between patient characteristics and survival with hazard ratios (HRs). Statistical tests were two‐sided, and *p* < 0.05 was considered significant. IBM SPSS Statistics 25 (IBM), R v4.0.5 (R Development Core Team) and the R package MatchIt were used for statistical analyses.

## 3 RESULTS

### 3.1 Propensity score matching

Before matching, the mean age of *PHF6* ^mut^AML patients was 46.27 (20–69) and 59% (*n* = 13) were male. The risk categories included favourable (*n* = 3, 14%), intermediate (*n* = 7, 32%) and poor (*n* = 12, 54%). What caught our attention was that more than half of the patients with *PHF6* mutation were assigned to the poor prognosis group, and only 38% of patients without *PHF6* mutation were assigned to the poor group. Thus, we constructed a propensity score‐matched analysis stratified by risk category as well as key baseline demographic characteristics (age at diagnosis and sex). All matched covariates had standardised mean differences <0.1 (Table 1), indicating excellent balance between matched covariates.^15^

**TABLE 1:** Patient characteristics before and after propensity score matching^a^

Characteristic	Before matching			After matching		
	*PHF6* ^mut^AML (*n* = 22)	*PHF6* ^wt^AML (*n* = 442)	*p* Value	*PHF6* ^mut^AML (*n* = 22)	*PHF6* ^wt^AML (*n* = 43)	Standardised mean difference
Age, mean (SD), y	46.27 (15.45)	44.92 (15.26)	0.998	46.27 (15.45)	46.12 (15.76)	0.0294
Sex			0.088			
Male	13 (59%)	247 (56%)		13 (59%)	27 (63%)	−0.0925
Female	9 (41%)	195 (44%)		9 (41%)	16 (37%)	0.0925
Risk Category^b^			0.071			
Favourable	3 (14%)	128 (29%)		3 (14%)	6 (14%)	0
Intermediate	7 (32%)	147 (33%)		7 (32%)	14 (33%)	0
Poor	12 (54%)	167 (38%)		12 (54%)	23 (53%)	0

Abbreviations: SD, standard deviation.

^a^ All percentages are based on the total number of patients in each cohort (*n*), unless specified. Continuous variables are presented as the mean (SD), and categorical variables are presented as number (percentage).

^b^ Risk categories were identified according to the ELN 2017 recommendation.

### 3.2 Baseline characteristics of the patients of matched cohorts

The clinical and laboratory characteristics of 22 *PHF6* ^mut^AML and 43 matched *PHF6* ^wt^AML patients are shown in Table 2. In terms of important clinical, cytogenetic and molecular features, *PHF6* mutation was significantly associated with *IDH2* mutation (27% in *PHF6* ^mut^ AML vs. 2% in *PHF6* ^wt^AML, *p* = 0.005), *KRAS* mutation (14% in *PHF6* ^mut^ AML vs. 0% in *PHF6* ^wt^AML, *p* = 0.035) and +11 (14% in *PHF6* ^mut^AML vs. 0% in *PHF6* ^wt^AML, *p* = 0.035), and no differences were found in the odds of having other prognosis‐related gene mutations and important chromosomal aberrations. In addition, we found no statistically significant differences in the choices of induction therapy or whether allo‐HSCT was performed.

**TABLE 2:** Clinical and laboratory characteristics of 22 *PHF6* ^mut^AML and 43 matched *PHF6* ^wt^AML patients^a^

Feature	*PHF6* ^mut^AML (*n* = 22)	*PHF6* ^wt^AML (*n* = 43)	*p* Value^b^
WBC (10^9^/L)	2.57 (1.73–38.29)	9.39 (2.79–59.83)	0.377
HB (g/L)	81.35 (20.23)	76.41 (22.83)	0.421
PLT (10^9^/L)	57.0 (37.25–84.25)	36.0 (18.5–72.0)	0.066
Bone marrow blasts, %	60.05 (20.54)	57.09 (22.45)	0.192
Chromosomal aberrations			
*t* (8; 21)	0	3 (7%)	0.545
Inv (16)	0	2 (5%)	0.545
+8	1 (5%)	4 (9%)	0.655
−7/7q‐	0	3 (7%)	0.545
+11	3 (14%)	0	**0.035**
Monosomal karoytype	0	5 (12%)	0.158
Complex karoytype	1 (5%)	5 (12%)	0.655
Molecular diagnostics			
*RUNX1*	2 (9%)	4 (9%)	1.0
*ASXL1*	7 (32%)	6 (14%)	0.109
*TP53*	2 (9%)	3 (7%)	1.0
*NPM1*	3 (14%)	4 (9%)	0.681
*FLT3‐ITD*	3 (14%)	5 (12%)	1.0
*IDH1*	3 (14%)	2 (5%)	0.326
*IDH2*	6 (27%)	1 (2%)	**0.005**
*DNMT3A*	4 (18%)	2 (5%)	0.168
*WT1*	2 (9%)	3 (7%)	1.0
*KRAS*	3 (14%)	0	**0.035**
*TET2*	5 (23%)	3 (7%)	0.108
*MLL* rerragement	2 (9%)	6 (14%)	0.706
Allo‐HSCT	7 (32%)	18 (42%)	0.591
Treatment Induction			0.658
Standard‐dose cytarabine based	13 (59%)	28 (65%)	
Low‐dose cytarabine based	8 (36%)	13 (30%)	
Hypomethylating agents only	1 (5%)	2 (5%)	

Abbreviations: +11, additional chromosome 11; +8, additional chromosome 8; −7/7q‐, delection of chromosome 7/7q; allo‐HSCT, allogeneic haematopoietic stem cell transplantation; HB, haemoglobin; Inv (16), inversion of chromosome 16; PLT, platelet count; *t* (8;21), translocation that involves chromosome 8 and chromosome 21; WBC, white blood cell count.

^a^ All percentages are based on the total number of patients in each cohort (n), unless specified. Datas are presented as number (percentage) for categorical variables, mean (SD) for normally distributed continuous variables and median (interquartile range) for nonnormally distributed continuous variables.

^b^ *p* values in bold are statistically significant; *p* < 0.05.

Given the role of *PHF6* in lineage plasticity, we further described the immunophenotypic characteristics of leukaemic blasts in *PHF6* ^mut^AML to explore whether these leukaemic blasts were accompanied by the expression of other lineage markers. In leukaemic blasts from *PHF6* ^mut^AML, the most common myeloid differentiation antigens were CD33 (100%, 20/20), CD117 (95%, 19/20), CD13 (83%, 15/18), MPO (65%, 11/17), CD64 (63%, 12/19) and CD15 (41%, 7/17). Nonlineage‐specific differentiation antigens included HLA‐DR (100%, 20/20), CD34 (95%, 19/20), CD38 (95%, 19/20), CD123 (94%, 17/18) and TdT (36%, 4/11). Additionally, aberrant expression of lymphoid antigens, including CD56 (41%, 7/17), CD7 (37%, 7/19), CD22 (15%, 2/13), CD19 (11%, 2/19) and cCD79a (7%, 1/15), was observed in some AML cases. cCD3, which is a powerful T‐lineage marker, was not detected in all cases.

### 3.3 Treatment responses and survival analysis of matched cohorts

First, the chemotherapy response was evaluated in patients with and without *PHF6* mutation. The CR rate was significantly lower in the *PHF6* ^mut^AML cohort than in the *PHF6* ^wt^AML cohort (41% vs. 69%; OR = 3.64, 95% CI 1.10, 12.10; *p* = 0.035). However, the rate of minimal residual disease (MRD) by flow cytometry in those patients who achieved CR was similar between the two groups (57% in *PHF6* ^mut^AML patients vs. 62% in *PHF6* ^wt^AML patients, *p* = 1.00) (Table 3). Additionally, the relapse rate and remission duration (*p* = 0.316) showed no significant difference between *PHF6* ^mut^AML and *PHF6* ^wt^AML patients.

**TABLE 3:** Response and survival outcomes of *PHF6* ^mut^AML and *PHF6* ^wt^AML patients^a^

Response outcomes	*PHF6* ^mut^AML	*PHF6* ^wt^AML	OR (95% CI) for *PHF6* ^mut^AML vs. *PHF6* ^wt^AML	*p* value
CR	7 (41%)	29 (69%)	3.64 (1.10, 12.10)	**0.035**
MRD negative	4 (57%)	18 (62%)	—	1
Relapse	5 (63%)	14 (38%)	0.64 (0.10, 4.16)	0.641
30‐day mortality	3 (14%)	0	0.01 (0, 223.0)	0.340
6‐month mortality	9 (41%)	4 (10%)	0.13 (0.03, 0.63)	**0.011**

Abbreviations: CI, confidence interval; CR, complete remission; MRD, minimal residual disease by flow cytometry; OR, odds ratio.

^a^ All percentages are based on the number of patients who can be evaluated at the corresponding time point in each cohort. The total number of CR patients was used as the denominator for relapse. The total number of CR patients were used as the denominator for the absence of MRD. Categorical data are presented as number (percentage). *p* values in bold are statistically significant; *p* < 0.05.

**FIGURE 2:**
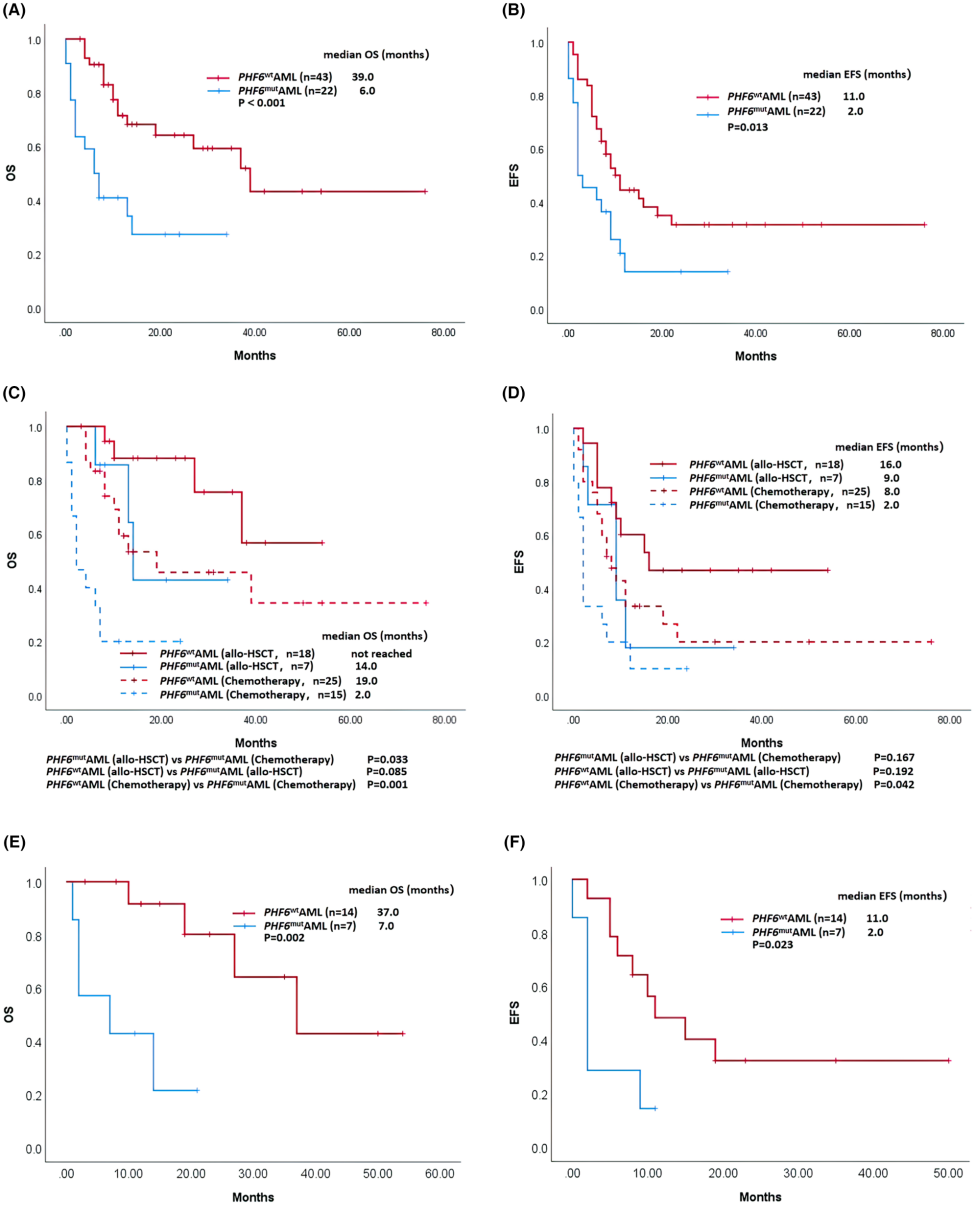
Kaplan–Meier survival curves comparing survival in different patients with AML. (A, B) Overall survival (OS) and event‐free survival (EFS) in patients with and without *PHF6* mutation. (C, D) OS and EFS in *PHF6* ^mut^AML and *PHF6* ^wt^AML patients stratified by different treatments (receiving allo‐HSCT or only chemotherapy). (E, F) OS and EFS in *PHF6* ^mut^AML and *PHF6* ^wt^AML patients who were assigned to the moderate prognosis group according to ELN 2017 risk categories

Second, we performed survival analysis. *PHF6* ^mut^AML patients had a significantly shorter median OS than *PHF6* ^wt^AML patients (6.0 vs. 39.0 months; *p* < 0.001) (Figure 2A), and the 1‐year OS rate among patients with *PHF6* mutation was much lower than that among patients without *PHF6* mutation (40.9% for *PHF6* ^mut^AML patients vs. 71.5% for *PHF6* ^wt^AML patients, *p* = 0.023). Similarly, the median EFS (2.0 vs. 11.0 months, *p* = 0.013) (Figure 2B) was shorter in *PHF6* ^mut^AML patients. There was no difference in median RFS (8.0 vs. 19.0 months; *p* = 0.664) between *PHF6* ^mut^AML and *PHF6* ^wt^AML patients.

The low CR rate and poor survival suggest that conventional chemotherapy was less effective in *PHF6* ^mut^AML patients, which may be partly due to drug resistance. To date, the mechanism of *PHF6* mutation‐mediated resistance in AML remains unknown; thus, further clinical and basic studies are urgently needed. Fortunately, allo‐HSCT may improve the prognosis of such patients. *PHF6* ^mut^AML patients who received allo‐HSCT had a prolonged median OS compared to those only treated with chemotherapy (14.0 vs. 2.0 months; *p* = 0.033), with no difference in EFS (9.0 vs. 2.0 months; *p* = 0.167) (Figure 2C,D). Additionally, *PHF6* ^mut^AML and *PHF6* ^wt^ AML patients who received allo‐HSCT showed comparable OS (14.0 months vs. not reached, *p* = 0.085) and EFS (9.0 vs. 16.0 months; *p* = 0.192), suggesting that allo‐HSCT may at least partly overcome the poor prognosis related to *PHF6* mutation (Figure 2C,D).

Considering the co‐mutation tendency of *IDH2* mutation and *KRAS* mutation with *PHF6* mutation, we also performed further survival analyses. The co‐mutation genotype did not show any difference in survival (OS, 4.0 months in *PHF6* ^mut^/*IDH2* ^mut^AML vs. 6.0 months in *PHF6* ^mut^/*IDH2* ^wt^AML, *p* = 0.604; EFS, 2.0 months in *PHF6* ^mut^/*IDH2* ^mut^AML vs. 2.0 months in *PHF6* ^mut^/*IDH2* ^wt^AML, *p* = 0.194) with respect to *IDH2* mutation. Additionally, survival outcomes for *PHF6* ^mut^/*KRAS* ^mut^AML seemed worse than those without *KRAS* mutation (OS, 2.0 months in *PHF6* ^mut^/*KRAS* ^mut^AML vs. 7.0 months in *PHF6* ^mut^/*KRAS* ^wt^AML, *p* = 0.036; EFS, 2.0 months in *PHF6* ^mut^/*KRAS* ^mut^AML vs. 6.0 months in *PHF6* ^mut^/*KRAS* ^wt^AML, *p* = 0.116). Aside from *IDH2* mutation and *KRAS* mutation, we also added analyses of the effects of other concomitant mutations on survival in *PHF6* ^mut^AML patients. These results showed that no other additional mutations were associated with clinical outcomes.

### 3.4 Impact of prognostic factors on survival

For all 65 AML patients, the median OS was 27.0 months. To identify all factors that influenced survival, univariate and multivariate analyses were subsequently performed on all patients in our study.

Univariate analysis revealed that +8 (*p* = 0.012), −7/7q‐ (*p* = 0.019), *PHF6* mutation (*p* < 0.001) and *KRAS* mutation (*p* < 0.001) were associated with reduced overall survival. Allo‐HSCT was associated with improved overall survival (*p* = 0.004). Characteristics significant in the univariate models at level 0.10 were included in the multivariate model. Multivariate analysis revealed that survival was adversely impacted by secondary AML (HR = 3.895, 95% CI 1.20, 12.63; *p* = 0.023), +8 (HR = 8.448, 95% CI 2.14, 33.42; *p* = 0.002), −7/7q‐ (HR = 7.318, 95% CI 1.28, 41.99; *p* = 0.026) and *PHF6* mutation (HR = 8.910, 95% CI 3.51, 22.63; *p* < 0.001). In contrast, treatment with allo‐HSCT (HR = 0.297, 95% CI 0.12, 0.74; *p* = 0.009) was a prognostic marker for improved OS.

### 3.5 Prognostic value of *PHF6* mutation in intermediate‐risk AML

In our study, there were 21 intermediate‐risk AML patients (7 *PHF6* ^mut^AML patients and 14 *PHF6* ^wt^AML patients) who were assigned to the moderate prognosis group according to the ELN 2017 risk categories. Similar to the results of matched‐cohort analysis, we also found that *PHF6* ^mut^AML patients had a significantly shorter median OS (7.0 vs. 37.0 months; *p* = 0.002) and median EFS (2.0 vs. 11.0 months; *p* = 0.023) than *PHF6* ^wt^AML patients in intermediate‐risk AML (Figure 2E,F).

For those patients assigned to the favourable prognosis group (low‐risk AML), *PHF6* mutation may also represent poor prognosis (OS, *p* = 0.027; EFS, *p* = 0.009) (Figure S1A,B). However, such results need to be further validated because of limited cases in the favourable prognosis group. In addition, among patients assigned to the poor prognosis group (high‐risk AML), there was no difference in survival between *PHF6* ^mut^AML and *PHF6* ^wt^AML patients (OS, *p* = 0.143; EFS, *p* = 0.559) (Figure S1C,D).

## 4 DISCUSSION

*PHF6* encodes a chromatin remodelling protein that is involved in a variety of biological processes including lineage plasticity within haematopoietic malignancies.^16^, ^17^, ^18^ Soto‐Feliciano et al. reported that *PHF6* played an important role in regulating chromatin accessibility to lineage‐specific transcription factors and that loss of *Phf6* could result in disruption of lineage differentiation. Moreover, loss of *Phf6* in leukaemia lymphoblasts activates a leukaemia stem cell transcriptional program and drives enhanced T‐ALL leukaemia‐initiating cell activity.^8^ To determine whether *PHF6* ^mut^AML has aberrant expression of lymphoid antigens, we have further described the immunophenotypic characteristics of leukaemic blasts. The results suggested that most of the leukaemic blasts in *PHF6* ^mut^AML still showed a typical myeloid blast immunophenotype, and some aberrant expression of lymphoid antigens, including 41% expressed CD56, 37% expressed CD7 and 11% expressed CD19. We reported a lineage differentiation profile similar to previous studies which reported that CD56 (26%–41%) and CD7 (21–42%) were the most commonly expressed lymphoid markers in AML, whereas the incidence of positivity for the B‐cell‐associated markers (approximately 10% for CD19) was low.^19^, ^20^, ^21^

In addition, *PHF6* is important for leukaemogenesis. Recent findings from *Phf6* conditional knockout mouse models showed that the loss of *Phf6* synergizes with leukaemic lesions, such as aberrant expression of TLX3, activating mutations in NOTCH1 or JAK3 activation to promote the onset of T‐ALL, demonstrating a tumour‐suppressive function for *PHF6* in T‐ALL.^7^, ^8^, ^9^, ^22^, ^23^ However, the potential role of *PHF6* in AML has seldom been studied, and the clinical features and prognostic implications of *PHF6* mutation in AML remain unclear. Thus, the clinical significance and biological function of *PHF6* in AML have yet to be clarified. Our propensity score‐matched cohort study which identified 22 patients with *PHF6* mutation from 801 AML patients contains the largest cases of *PHF6* mutation in AML reported to date. Importantly, our survival analyses showed that *PHF6* mutation was associated with inferior median EFS (2.0 vs. 11.0 months, *p* = 0.013) and median OS (6.0 vs. 39.0 months; *p* < 0.001) compared with *PHF6* wild‐type patients. In addition, the results of univariate analysis and the subsequent multivariate analysis consistently supported that *PHF6* mutation was associated with an adverse outcome. Similar results were obtained when the analysis was restricted to intermediate‐risk AML patients. These results were consistent with a previous study by Patel et al., which reported that *PHF6* mutation was associated with adverse prognosis via univariate analysis.^6^ Gaidzik et al. also reported a lower median OS (6.0 months in *PHF6* ^mut^ AML vs. 16.0 months in *PHF6* ^wt^AML; *p* = 0.03) among AML patients habouring *RUNX1* mutation.^24^

It is known that allo‐HSCT could be an effective therapeutic in intermediate and high‐risk AML. We also found that receipt of allo‐HSCT was correlated with improved median OS compared with those who did not receive allo‐HSCT in *PHF6* ^mut^AML patients. In addition, allo‐HSCT in the first CR resulted in similar survival to that of patients with wild‐type *PHF6*. Thus, our study may provide some information for strategy decision making. Although patients in the intermediate prognosis group can either receive chemotherapy or allo‐HSCT for consolidation according to AML guidelines, our data suggested that *PHF6* may be related to poor prognosis and that it may be better to perform allo‐HSCT for *PHF6* ^mut^AML patients assigned to the intermediate prognosis group. Even for *PHF6* ^mut^AML patients in the favourable prognosis group, more close monitoring of MRD during consolidation and after the end of therapy may be necessary.

Our data showed a trend for a shorter RFS in *PHF6* ^mut^AML (8.0 vs. 19.0 months; *p* = 0.664), although there were no significant differences in RFS or remission duration, which was partly due to the relatively small sample size. Besides, 63% of *PHF6* ^mut^AML patients experienced relapse. As a matter of fact, in our AML cases, we used intermediate‐dose cytarabine (IDAC) for consolidation therapy. It was reported that high‐dose cytarabine (HiDAC) may lower relapse risk compared with IDAC for consolidation in favourable‐risk AML.^25^ In addition, according to a meta‐analysis comparing the efficacy of different post‐remission cytarabine doses, HiDAC showed better disease‐free survival in those patients with favourable cytogenetics, but this did not translate into an OS benefit.^26^ Considering the extremely poor outcomes in *PHF6* ^mut^AML, whether HiDAC can reduce relapse or improve survival in *PHF6* ^mut^AML patients needs to be clarified in the further studies.

What is more, our study reported a lower CR rate in the *PHF6* ^mut^AML cohort than in the *PHF6* ^wt^AML cohort (41% vs. 69%; OR = 3.64, 95% CI 1.10, 12.10; *p* = 0.035). The data were consistent with Gaidzik et al.'s study in which the data of 246 AML patients with *RUNX1* mutation were analysed. Among patients with different additional mutations, the chemotherapy response was lowest for patients with a genotype of *RUNX1* ^mut^/*PHF6* ^mut^ (22.2% in *RUNX1* ^mut^/*PHF6* ^mut^ patients vs. 60.9% in *RUNX1* ^mut^/*PHF6* ^wt^ patients; *p* = 0.03).^24^ These results suggested that patients with *PHF6* mutation showed more chemotherapy resistance.

It was reported that *PHF6* mutation mediated drug resistance in T‐ALL. Depletion of *PHF6* decreased the drug sensitivity of T‐ALL to prednisolone by repressing p21 expression, and cotreatment with a p21 inhibitor increased the sensitivity of T‐ALL cells to prednisolone.^27^ However, the mechanism of *PHF6*‐mediated drug resistance in AML remains unexplored. Our team found that significantly elevated levels of pre‐rRNA were observed in clinical AML patients with *PHF6* mutation and loss of *PHF6* may promote rDNA transcription to mediate chemotherapy resistance by regulating histone epigenetic modification of rDNA loci (data not shown). We believe that further detailed mechanistic studies may help to identify novel therapeutic targets for *PHF6* mutant AML patients.

Finally, although propensity score matching could provide an effective way to minimise bias, our study still has some limitations due to its retrospective and single‐centre nature. Moreover, the number of enrolled cases was relatively small. These findings need to be validated in large, homogeneously treated, multicentre cohorts of patients with AML in the future.

In summary, we performed a propensity score matching study for *PHF6* mutant and wild‐type AML patients in our AML cohort from a series of 801 cases and identified 22 *PHF6* ^mut^AML cases and 43 matched *PHF6* ^wt^AML cases. Our data demonstrated that patients with AML harbouring *PHF6* mutation had a lower CR rate to induction chemotherapy and shorter overall survival. Furthermore, *PHF6* ^mut^AML and *PHF6* ^wt^AML patients who received allo‐HSCT showed comparable OS. Our study suggests that *PHF6* mutation may be related to poor prognosis, and the detection of *PHF6* mutation in AML would tend to support the selection of more active treatments.

## AUTHOR CONTRIBUTIONS

Kexiu Huang and Rui Huang contributed to the study design, Kexiu Huang, Lei Wang, Yaling Zheng and Chunyan Yue were involved in the collection and assembly of clinical data. Kexiu Huang and Xuedan Xu performed data analysis and wrote the manuscript. Hongbo Chen, Rui Huang and Yuhua Li supervised the study.

## FUNDING INFORMATION

This work was supported by the National Natural Science Foundation of China (grant number: 82100157 ; U2001224; 81970145) and Natural Science Foundation of Guangdong Province, China (grant number: 2020A1515011465).

## CONFLICT OF INTEREST

The authors declare no conflicts of interest.

## ETHICAL APPROVAL STATEMENT

The study was approved by the Ethics Committees of Zhujiang Hospital of Southern Medical University.

## Supporting information


Figure S1

## Data Availability

The data that support the findings of this study are available on request from the corresponding author. The data are not publicly available due to privacy or ethical restrictions.
