# Supplementary material for: PHF6 mutation is associated with poor outcome in acute myeloid leukaemia
Source: Cancer Med. 2022 Sep 29;12(3):2795–804. doi: 10.1002/cam4.5173 (PMC9939093; doi:10.1002/cam4.5173)
Supplement: Supplementary file 1 — Figure S1 [file CAM4-12-2795-s001.pdf]

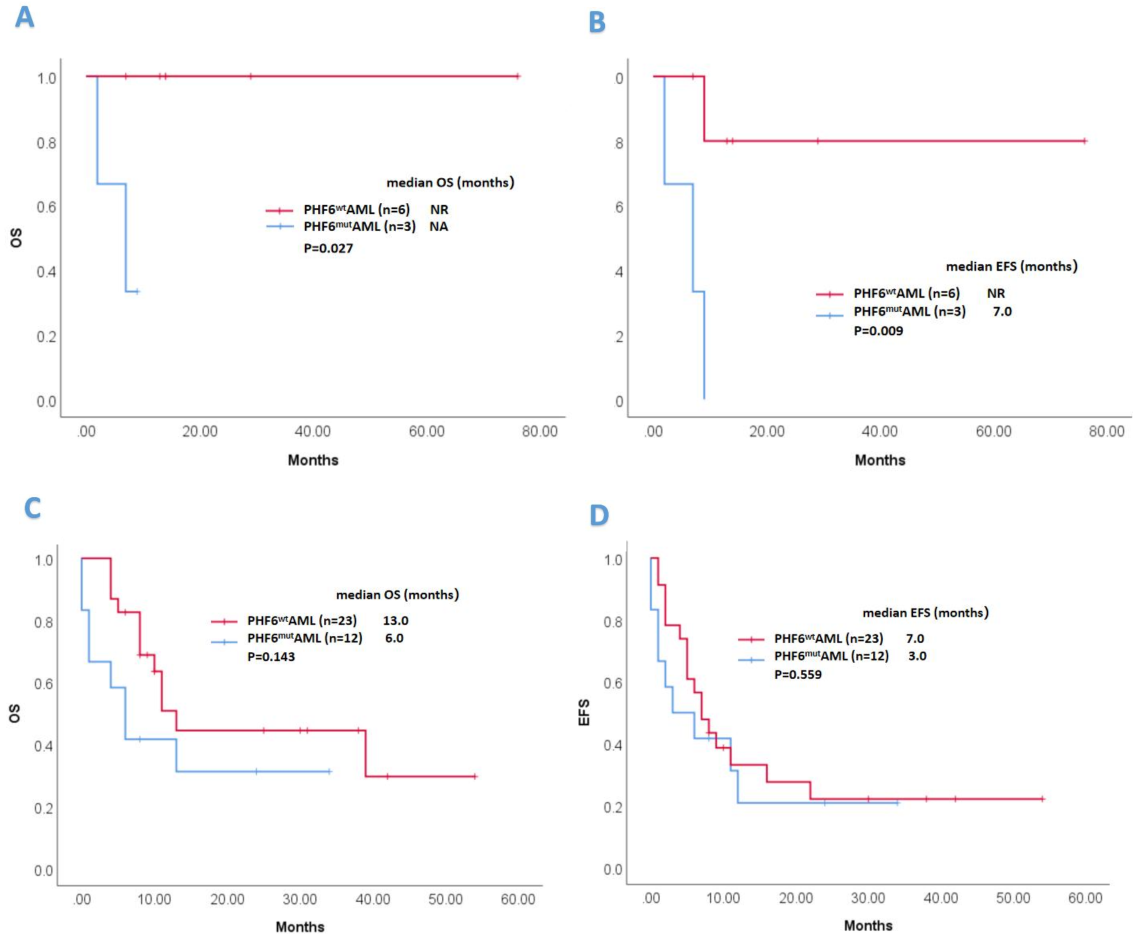

Supplementary Figure S1. Kaplan-Meier survival curves comparing survival in different patients with AML.

Abbreviations: OS, overall survival; EFS, event-free survival; NR, not reached; NA, not available.

A-B: OS and EFS in PHF6^{mut}AML and PHF6^{wt}AML patients who were assigned to the favourable prognosis group according to ELN 2017 risk categories. C-D: OS and EFS in PHF6^{mut}AML and PHF6^{wt}AML patients who were assigned to the poor prognosis group.
